# Supplementary material for: The role of microglial LRRK2 kinase in manganese-induced inflammatory neurotoxicity via NLRP3 inflammasome and RAB10-mediated autophagy dysfunction
Source: J Biol Chem. 2023 Jun 1;299(7):104879. doi: 10.1016/j.jbc.2023.104879 (PMC10331485; doi:10.1016/j.jbc.2023.104879)
Supplement: Supporting Information S1 [file mmc1.docx]

**
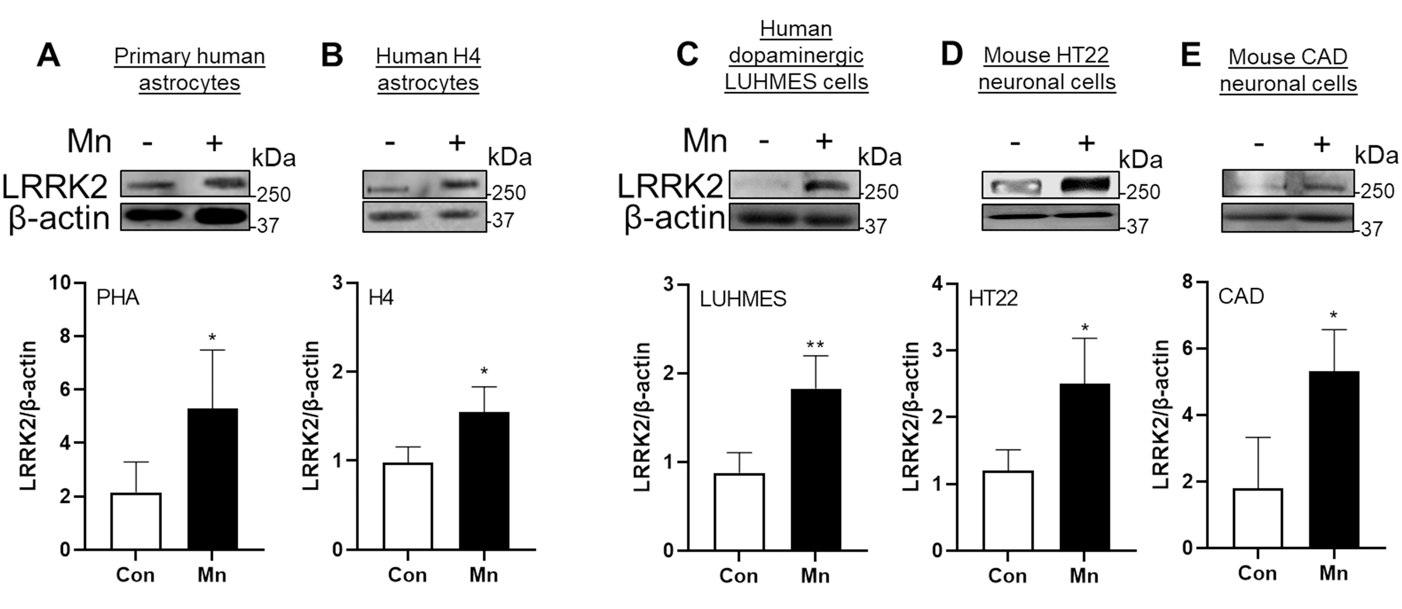
**

**Supporting Information 1. Mn increases LRRK2 in astrocytes and neuronal cell cultures.** Astrocyte and neuronal cells were prepared and cultured in 6-well plates, followed by Mn exposure (250 μM, 12 h). Mn increases LRRK2 protein levels in various cell types, including primary human astrocytes (A) and human H4 astrocytes (B), a differentiated human Lund mesencephalic (LUHMES) dopaminergic cells (C), mouse HT22 hippocampal neuronal cells (D), and mouse cath.a differentiated (CAD) catecholaminergic neuronal cells (E). β-actin was used as a loading control. ^*^p < 0.05, ^**^p < 0.01, compared with the controls. Data is expressed as mean ± SD (unpaired Student’s *t*-test, n = 3). The data shown are representative of three independent experiments.
